# Supplementary material for: The role of oxygen intake and liver enzyme on the dynamics of damaged hepatocytes: Implications to ischaemic liver injury via a mathematical model
Source: PLoS One. 2021 Apr 22;16(4):e0230833. doi: 10.1371/journal.pone.0230833 (PMC8061939; doi:10.1371/journal.pone.0230833)
Supplement: S1 File — (PDF) [file pone.0230833.s001.pdf]

S1 File
Data Extraction
The data used in this paper is presented in the excel document. AST, ALT and LDH values were extracted from six different papers. Two of those papers were used to estimate the parameters $\beta_S, \beta_L, \beta_D, D_{max}$. These two papers were authored by Raurich [34] and Henrion [20]. From [34] the peak AST, ALT, and LDH values, as well as their range are extracted from Table 2. Sheet 5 of the excel spreadsheet, labeled Raurich displays all the tables that provide information on the 182 patients from which the serum values were calculated. The values taken are shown in the Raurich subsection of the first excel sheet in the document. Table 1 provides clinical characteristics such as age, sex, weight, causes of hypoxic hepatitis, and underlying conditions. Tables 2 and Table 3 display laboratory data such as serum levels and blood gas analysis results. Table 4 presents statistics on the treatment the patients received, and Table 5 displays factors that may have affected mortality.

The other paper used for parameter estimation is that by Henrion [20]. From [20], rows from two columns of Table 2 are extracted. These columns present peak AST, ALT, and LDH data for Ischaemic Hepatitis resulting from the conditions acute cardiac failure (ACF) and congestive heart failure (CHF), both of which fall under our scope of interest. Sheet 3 of the excel document presents the tables [20]. The second and third columns of the tables discuss these cases. Table 1 discusses clinical findings of the groups. Table 2 and Table 3 present laboratory and hemodynamic data. Tables 4 and 5 show results of a hemodynamic assessment and measurements of hepatic blood flow for these groups. Tables 6 and 7 describe the clinical and laboratory data sets from which their analysis was made. This includes the numbers of cases, gender, age, causes of hypoxia, and their associated serum values. The Raurich [34] paper and the two sections from the Henrion[20] paper provide three values for each serum (AST, ALT, and LDH). These three values are averaged to estimate the aforementioned parameters.

To validate the model, the numbers are extracted from the four other articles. Three numbers for each serum (AST, ALT, and LDH) came from Birrer [4]. These nine numbers came from the paper's third table for cases of decreased oxygen (Group 1), decreased oxygen delivery (Group 2), and decreased oxygen availability (Group 3). In the second sheet of the excel document, this table, as well as the other [4] are attached. The first table provides data readings such as cardiac index, central venous pressure, and hepatic blood flow. The second table provides statistics on the conditions that caused the diagnosis of hypoxia. The third table, from which our information was acquired, provides the ages, serum levels, and laboratory data for the three groups used.

From the Drolz [10], four values are extracted from the text, two for AST and two for ALT. These values represent average peak serum values for patients with and without statin therapy treatment. The values themselves are found written in the manuscript's outcomes section which is in the fourth sheet of the excel document. Details such as mortality, complications, and creatinine concentrations were also found in the text. Tables 1 and 2 provide the associated information regarding the number of patients, ages, causes, outcomes, and treatment administered.

The Tapper [40] provide a table (Table 2) that include AST and ALT peak values. In order to extract a single AST and a single ALT value, the columns of the table are averaged. In the Tapper section of the excel spreadsheet, details about the sources of their data can be found. In Table 1,

this information includes the years and authors of the data. It also includes the sample size and number of ischaemia cases. In Table 2, the serum values can be found, as well as the percentage of hypotensive events, cardiac events, sepsis, and survival rates.

Henrion's[18], presents AST, ALT, and LDH values in Table 7. Once again, the peak values are averaged such that only one value for each serum is taken from this paper. In the last sheet of the excel document, the tables from [18] can be found. The first two tables provide data on patient hospital and intensive care unit admittance. Table 3 details the underlying conditions that contributed to the hypoxic hepatitis cases. Tables 4-6 detail hemodynamic data associated with each underlying condition such as oxygen delivery and cardiac index. Table 7, from which the values were extracted, contains data about the number of cases, age, sex, survival, and serum levels.

The resulting values were compiled in a table which can be found at the bottom of the first sheet in the excel document. These nine AST, nine ALT, and five LDH values not used in parameter estimation are averaged. The resulting average values are compared with the peak values from our mathematical model for validation. This analysis weighed data from individual papers or sections of papers equally and only constrained the data in cases where decreased oxygen or heart conditions are explicitly differentiated in the sources. The data analysis did not consider patient numbers, demographic information, or the severity of a patient's condition.
