# Supplementary material for: The role of oxygen intake and liver enzyme on the dynamics of damaged hepatocytes: Implications to ischaemic liver injury via a mathematical model
Source: PLoS One. 2021 Apr 22;16(4):e0230833. doi: 10.1371/journal.pone.0230833 (PMC8061939; doi:10.1371/journal.pone.0230833)
Supplement: Attachment — Submitted filename: Response-letter-Ischaemic.pdf [file pone.0230833.s003.pdf]

Letter of Response to Reviewer's comments

The mapped response to editor's comments (numbered from 1 through 6) and reviewer's comments (numbered from 1 through 5) are given below. Our response to the comments (in italics below) is followed by a short description of what has been added to the paper.

Response to Editor

1. *Please ensure that your manuscript meets PLOS ONE's style requirements, including those for file naming.* https://journals.plos.org/plosone/s/file?id=wjVg/PLOSOne_formatting_sample_main_body.pdf

https://journals.plos.org/plosone/s/file?id=ba62/PLOSOne_formatting_sample_title_authors_affiliations.pdf

Response-We have adopted the PLOS ONE style template and have formatted our article according to the requirements of PLOS ONE.

2. *Thank you for including your funding statement; "none". At this time, please address the following queries:*

a. *Please clarify the sources of funding (financial or material support) for your study. List the grants or organizations that supported your study, including funding received from your institution.*

   Response- We did not receive funding from any organization.

b. *State what role the funders took in the study. If the funders had no role in your study, please state: "The funders had no role in study design, data collection and analysis, decision to publish, or preparation of the manuscript."*

   Response-The funders had no role in study design, data collection and analysis, decision to publish, or preparation of the manuscript.

c. *If any authors received a salary from any of your funders, please state which authors and which funders.*

   Response-The authors did not receive any salary for this work.

d. *If you did not receive any funding for this study, please state: "The authors received no specific funding for this work."*

   Response-The authors received no specific funding for this work.

e. *Please include your amended statements within your cover letter; we will change the online submission form on your behalf.*

   Response-We will indicate our funding status in the cover letter in details.

3. *Thank you for including your competing interests statement; "No"*

Please complete your Competing Interests on the online submission form to state any Competing Interests. If you have no competing interests, please state "The authors have declared that no competing interests exist.", as detailed online in our guide for authors at http://journals.plos.org/plosone/s/submit-now

This information should be included in your cover letter; we will change the online submission form on your behalf.

Response-Thank you, we will include in our cover letter that the authors have declared that no competing interests exist.

4. In your Data Availability statement, you have not specified where the minimal data set underlying the results described in your manuscript can be found. PLOS defines a study's minimal data set as the underlying data used to reach the conclusions drawn in the manuscript and any additional data required to replicate the reported study findings in their entirety. All PLOS journals require that the minimal data set be made fully available. For more information about our data policy, please see http://journals.plos.org/plosone/s/data-availability.

Upon re-submitting your revised manuscript, please upload your study's minimal underlying data set as either Supporting Information files or to a stable, public repository and include the relevant URLs, DOIs, or accession numbers within your revised cover letter. For a list of acceptable repositories, please see http://journals.plos.org/plosone/s/data-availability#loc-recommended-repositories. Any potentially identifying patient information must be fully anonymized.

Important: If there are ethical or legal restrictions to sharing your data publicly, please explain these restrictions in detail. Please see our guidelines for more information on what we consider unacceptable restrictions to publicly sharing data: http://journals.plos.org/plosone/s/data-availability#loc-unacceptable-data-access-restrictions. Note that it is not acceptable for the authors to be the sole named individuals responsible for ensuring data access.

We will update your Data Availability statement to reflect the information you provide in your cover letter.

Response-The data used in this article is included in an excel document which contains five sheets as Supplementary Information 1 and a detailed explanation of the sheets as Supplementary Information 2. We have put all the tables from previously published papers that have been used for estimation in this article, in the excel sheets. We will submit the excel document and the explanation as supplementary information during submission. Below, we also provide a detailed explanation of the data in the excel sheets which we will attach during submission.

AST, ALT and LDH values are extracted from six different papers. Two of those papers are used to estimate the parameters $\beta_S, \beta_L, \beta_D, D_{max}$. These two papers are authored by Raurich [34] and Henrion [20]. From [34] the peak AST, ALT, and LDH values, as well as their range were extracted from Table 2. Sheet 5 of the excel spreadsheet, labeled Raurich displays all the tables from [34] that provide information on the 182 patients from which the serum values were calculated. The values taken are shown in the Raurich subsection of the first excel sheet in the document. Table 1 provides clinical characteristics such as age, sex, weight, causes of hypoxic hepatitis, and underlying conditions. Tables 2 and Table 3 display laboratory data such as serum levels and blood gas analysis results. Table 4 presents statistics on the treatment the patients received, and Table 5 displays factors that may have affected mortality.

The other article used for parameter estimation is Henrion [20]. From [20], rows from two columns of Table 2 are extracted. These columns present peak AST, ALT, and LDH data for Ischaemic Hepatitis resulting from the conditions of acute cardiac failure (ACF) and congestive heart failure (CHF), both of which fall under our scope of interest. Sheet 3 of the excel document presents the tables from [20]. The second and third columns of the tables discuss these cases. Table 1 discusses clinical findings of the groups. Table 2 and Table 3 present laboratory and hemodynamic data. Tables 4 and 5 show results of a hemodynamic assessment and measurements of hepatic blood flow for these groups. Tables 6 and 7 describe the clinical and laboratory data sets from which their analysis was made. This includes the numbers of cases, gender, age, causes of hypoxia, and their associated serum values. The Raurich [34] paper and the two sections from the Henrion [20] paper provide three values for each serum (AST, ALT, and LDH). These three values are averaged to estimate the aforementioned parameters.

To validate the model, the numbers are extracted from four other articles. The data for each serum (AST, ALT, and LDH) came from Birrer [4] from the third table for cases of decreased oxygen (Group 1), decreased oxygen delivery (Group 2), and decreased oxygen availability (Group 3). In the second sheet of the excel document, this table, as well as the others from the paper are attached. The first table provides data readings such as cardiac index, central venous pressure, and hepatic blood flow. The second table provides statistics on the conditions that caused the diagnosis of hypoxia. The third table, from which our information is acquired, provides the ages, serum levels, and laboratory data for the three groups used.

From Drolz [10], four values are extracted from the text, two for AST and two for ALT. These values represent average peak serum values for patients with and without statin therapy treatment. The values themselves are found written in the manuscript's outcomes section which is in the fourth sheet of the excel document. Details such as mortality, complications, and creatinine concentrations were also found in the text. Tables 1 and 2 provide the associated information regarding the number of patients, ages, causes, outcomes, and treatment administered.

Tapper [40], provides a table (Table 2) that includes AST and ALT peak values. In order to extract a single AST and a single ALT value, the columns of the Table 2 are averaged. In the Tapper section of the excel spreadsheet, details about the sources of their data can be found. In Table 1, this information includes the years and authors of the data. It also includes the sample size and number of ischaemia cases. In Table 2, the serum values can be found, as well as the percentage of hypotensive events, cardiac events, sepsis, and survival rates.

Henrion's [18] paper, present AST, ALT, and LDH values in Table 7. Once again, the peak values are averaged such that only one value for each serum is taken from this article. In the last sheet of the excel document, the tables from this article [18] can be found. The first two tables provide data on patient hospital and intensive care unit admittance. Table 3 details the underlying conditions that contributed to the hypoxic hepatitis cases. Tables 4-6 detail hemodynamic data associated with each underlying condition such as oxygen delivery and cardiac index. Table 7, from which the values were extracted, contains data about the number of cases, age, sex, survival, and serum levels.

The resulting values are compiled in a table which can be found at the bottom of the first sheet in the excel document. These nine AST, nine ALT, and five LDH values are averaged and the resulting average values are compared with the peak values from our mathematical model for data validation. This analysis weighed data from articles that constrained cases with decreased oxygen or heart conditions. The data analysis did not consider patient numbers, demographic information, or the severity of a patient's condition. In the future, a more rigorous data analysis can be done by

averaging with respect to the number of patients in each reference's data set, rather than weighing each article's numbers equally.

5. Please remove your figures from within your manuscript file, leaving only the individual TIFF/EPS image files, uploaded separately. These will be automatically included in the reviewers' PDF.

Response-The figures are removed from the manuscript file.

6. Please ensure that you refer to Figure 2 in your text as, if accepted, production will need this reference to link the reader to the figure.

Response-We have referred figure 2 in the manuscript.

Response to Reviewer#1

1. *The way that this paper was written is rather unusual, and I would suggest that the authors to make substantial revisions so that the format of the manuscript is consistent with others published in this journal.*

   Response- We have formatted the paper according to PLOS template

   https://journals.plos.org/plosone/s/file?id=wjVg/PLOSOne_formatting_sample_main_body.pdf

   https://journals.plos.org/plosone/s/file?id=ba62/PLOSOne_formatting_sample_title_authors_affiliations.pdf

2. *The authors mentioned that they only considered one form of cell death, apoptosis, caused by multiple cytokines. The authors ignored an important pathological process, cytokine storm, which is a physiological reaction in humans and other animals in which the innate immune system causes an uncontrolled and excessive release of cytokines. Normally, cytokines are part of the body's immune response, but their sudden release in large quantities can cause multi-organ failure and eventually death. Because of cascade and positive feedback, many cytokines accumulate in damaged organs in a short time. So, without taking these factors into consideration, the significance of the model that the author constructed is rather questionable.*

   Response- As mentioned in the reviewer's comment, we indeed only considered one form of cell death. However, the reason for consideration of only single form of the cell death is based on and strongly supported by literature. Please consider my following response. We consider on **necrosis** and did not include apoptosis **at this time**. The focus of our present study is on ischemia/hypoxia caused by a decrease in oxygenated blood flow due to heart failure. The primary reason consistently cited in the literature for ischaemia due to heart failure is cell death due to necrosis. According to the 2003 paper [20], ischaemic hepatitis was originally defined in 1979 as "liver injury characterized by a centrilobular liver cell necrosis with a sharp increase in serum aminotransferase activity in the setting of cardiac failure." A similar definition is given in the 2011 article [34], where Raurich et al. where ischaemic hepatitis is defined as "a liver injury characterized by a centrilobular liver cell necrosis with a rapid increase in serum aminotransferases." The Kavoliuniene et al. (2012) [25] also describes cell death due to necrosis during ischaemic hepatitis due to cardiocirculatory failure. We consider our modeling assumptions based on data collected by Kavoliuniene [25] et

al. (2012) and Raurich et al.[34] (2001), who did not mention apoptosis or cytokines as a reason for cell death. The studies by Tapper et al. [40] and by Birrer et al. [4] provide further literature examples where necrosis is the only form of cell death attributed to this condition.

During the research process, we did not come across the process of cytokine storm as a contributing factor to ischaemic hepatitis resulting from heart failure. While cytokine storm is an important process, it is not related to the focus of our current work. We did not model liver injury due to immune responses and cytokine release, but rather due to a lack of oxygen and insufficient ATP. In the literature, there is some disagreement on the role of apoptosis as a cell death mechanism in this instance. While many sources, such as those mentioned above, define liver ischaemia solely in terms of necrosis, others suggest that apoptosis may play a role. For example, the 2011 paper *Hypoxic hepatitis* by Jean Henrion [18] mentions a debate on the type of cell death involved and that "recent studies have reported evidence for the role of apoptotic cell death in addition to necrotic (oncotic) cell death." Despite this, the inclusion of apoptosis does not yet appear widely accepted within the literature. For instance, newer articles by Aboelsoud et al. in 2017[1], and by Waseem et al. in 2016 [43] still characterize hypoxic hepatitis as a result of necrosis. For this reason, we only considered necrosis in the model.
In the future, we plan to extend this model to focus on reasons other than heart failure, where cell death due to apoptosis will become significant. Future inclusion of apoptosis in the model would then lead to the consideration of cytokines as suggested by the reviewer. This may also provide a chance to compare the contribution of each type of cell death to patient data. It will also help us model and evaluate reperfusion better.

3. *Although the authors also mentioned in the discussion that the consideration of apoptosis is too idealistic. Perhaps the inclusion of inflammatory cytokines such as IL-6 and TNF-α into the model would make it more convincing.*

   Response-In this manuscript, as mentioned in the previous response, we did not model ischaemic liver injury due to immune responses and cytokine release, but rather due to a lack of oxygen and insufficient ATP as suggested consistently in the literature. This area is liver ischemia/hypoxia caused by a decrease in oxygenated blood flow due to heart failure. We chose to focus our model on **necrosis** as ischaemic liver injury due to heart failure is characterized by a centrilobular liver cell necrosis with a rapid increase in serum aminotransferases. Hence, we do not include inflammatory cytokines in our model for ischaemic hepatitis due to cardiocirculatory failure.

4. *The sources of data were not described clearly. Critical information such as the number of patients, study locations, pathological degrees of the patients, as well as the demographic information of the patients (age, gender, etc) needs to be clearly provided, as they determine the validity of the models and many of them may be confounding factors for the calculations.*

   Response- We have now elaborated the enclosed as a Supplementary document file 1 (excel document containing 5 sheets) which includes all the relevant data that we have used in the present study. We also provide an explanation of these data in the 2nd Supplementary document file (as a pdf file). The details of the inclusion of data is provided in editor's response#4.

5. *A better understanding and model of apoptosis will help improve our reperfusion model.*

Response-We are unclear about specific question raised here, as this is a line from our manuscript. However, as we have mentioned in our previous response that we model cell death due to necrosis as ischaemic liver injury due to heart failure is characterized by a centrilobular liver cell necrosis. The incorporation of apoptosis into the model is left as future work.
