# Supplementary material for: The role of oxygen intake and liver enzyme on the dynamics of damaged hepatocytes: Implications to ischaemic liver injury via a mathematical model
Source: PLoS One. 2021 Apr 22;16(4):e0230833. doi: 10.1371/journal.pone.0230833 (PMC8061939; doi:10.1371/journal.pone.0230833)
Supplement: Attachment — Submitted filename: Review Report.pdf [file pone.0230833.s004.pdf]

Report on “The Role of Oxygen Intake and Liver Enzyme on the Dynamics of Damaged Hepatocytes: Implications to Ischaemic Liver Injury via a Mathematical Model

In this paper, authors presented a mathematical model to study the dynamics of ischaemic hepatitis at molecular level and calibrated the model with the published data available in literature. The stability analysis of equilibrium points was investigated. Numerical simulations were performed exhaustively. The paper is well written, clear and contains interesting results. There are minor comments:

- page 1, the title, The Dynamics → the Dynamics and A Mathematical Model → a Mathematical Model
- revise materials and methods section to include methods and software used to solve ODE system together with the calibration and estimation of parameters.
- line 74, on above goals → on the above goals
- line 161, future studies To model → future studies. To model
- line 212, in terms on the model → in terms of the model
- line 216, Eq.8 → Eq. 8 also check line 303, 305 for the same.
- line 306, with respect to ρ and k. → with respect to ρ and k
- line 352, fig → figs.
- line 410, In order to model the damage due to reperfusion we → In order to model the damage due to reperfusion, we
- line 426, do not → does not
- revise references as per the journal’s requirement e.g. “Birrer, R., Yasuharu, T., AND Tsuyoshi, T.”
- line 667, expressions terms → expressions in terms
- line 671, mention the names of variables.
- lines 675, 678, 681, 685, 50 % → 50%
- lines 688, 690, O2 → O_2
- revise figure 2 to include the rates of reactions and replace, LDH (L) → LDH (D).
- mention initial conditions for state variables in the figs caption.
- Figs 9a, 9b and 10 are missing and figs 1, 2 and 4 are repeated.
- In Table 1, Value → Unit
