# Supplementary material for: The role of oxygen intake and liver enzyme on the dynamics of damaged hepatocytes: Implications to ischaemic liver injury via a mathematical model
Source: PLoS One. 2021 Apr 22;16(4):e0230833. doi: 10.1371/journal.pone.0230833 (PMC8061939; doi:10.1371/journal.pone.0230833)
Supplement: Attachment — Submitted filename: Letter of Response to Reviewer-3.pdf [file pone.0230833.s005.pdf]

Letter of Response to Reviewer's comments

The mapped response to editor's comments and reviewer's comments are given below. Our response to the comments (in italics below) is added to the paper.

Response to Editor

1. *page 1, the title, The Dynamics $\rightarrow$ the Dynamics and A Mathematical Model $\rightarrow$ a Mathematical Model*

   Response-We have made the correction.

2. revise materials and methods section to include methods and software used to solve ODE system together with the calibration and estimation of parameters.

   Response- We have used Python to solve the system of ODE and for estimation of parameters which we have included in our manuscript.

3. *line 74, on above goals $\rightarrow$ on the above goals*

   Response- We have made the correction.

4. *line 161, future studies to model $\rightarrow$ future studies. To model*

   Response- We have made the correction.

5. *line 212, in terms on the model $\rightarrow$ in terms of the model*

   Response- We have made the correction.

6. *line 216, Eq.8 $\rightarrow$ Eq. 8 also check line 303, 305 for the same.*

   Response- We have made the correction.

7. *line 306, with respect to r and k. $\rightarrow$ with respect to r and k*

   Response- We have made the correction.

8. *line 352, fig $\rightarrow$ figs.*

   Response- We have made the correction.

9. *line 410, In order to model the damage due to reperfusion we $\rightarrow$ In order to model the damage due to reperfusion, we*

   Response- We have made the correction.

10 *line 426, do not → does not*

Response- We have made the correction.

11 *revise references as per the journal's requirement e.g. "Birrer, R., Yasuharu, T., AND Tsuyoshi, T."*

Response- We have used the PLOSONE style for references.

12 *line 667, expressions terms → expressions in terms*

Response- We have made the correction.

13 *line 671, mention the names of variables.*

Response- We have added the variables.

14 *lines 675, 678, 681, 685, 50 % → 50%*

Response- We have made the correction.

15 *lines 688, 690, O2 → O_2*

Response- We have made the correction.

16 *revise figure 2 to include the rates of reactions and replace, LDH (L) →LDH (D).*

Response- We have revised figures and made the correction.

17 *mention initial conditions for state variables in the figs caption.*

Response- We have put the initial conditions for state variables in the figs caption.

18 *Figs 9a, 9b and 10 are missing and figs 1, 2 and 4 are repeated.*

Response- We have added all the figures.

19 *In Table 1, Value → Unit*

Response- We have made the correction.
